# Flower color modification in *Torenia fournieri* by genetic engineering of betacyanin pigments

**DOI:** 10.1186/s12870-024-05284-1

**Published:** 2024-06-27

**Authors:** Masahiro Nishihara, Akiko Hirabuchi, Takuya Teshima, Shota Uesugi, Hideyuki Takahashi

**Affiliations:** 1 Iwate Biotechnology Research Center, 22-174-4 Narita, Kitakami, 024-0003 Iwate Japan; 2 Department of Bioscience and Biotechnology, Fukui Prefectural University, 4-1-1 Kenjojima, Matsuoka, Eiheiji-cho, Fukui, 910-1195 Japan; 3 Present Address: Department of Agriculture, School of Agriculture, Tokai University, 871-12 Sugidou, Mashikimach, Kamimashiki-gun, Kumamoto, 861-2205 Japan

**Keywords:** Betalain, Betacyanin, Genetic engineering, *Torenia fournieri*, Flower color

## Abstract

**Background:**

Betalains are reddish and yellow pigments that accumulate in a few plant species of the order Caryophyllales. These pigments have antioxidant and medicinal properties and can be used as functional foods. They also enhance resistance to stress or disease in crops. Several plant species belonging to other orders have been genetically engineered to express betalain pigments. Betalains can also be used for flower color modification in ornamental plants, as they confer vivid colors, like red and yellow. To date, betalain engineering to modify the color of *Torenia fournieri—*or wishbone flower—a popular ornamental plant, has not been attempted.

**Results:**

We report the production of purple-reddish-flowered torenia plants from the purple torenia cultivar “Crown Violet.”  Three betalain-biosynthetic genes encoding CYP76AD1, dihydroxyphenylalanine (DOPA) 4,5-dioxygenase (DOD), and *cyclo*-DOPA 5-*O*-glucosyltransferase (5GT) were constitutively ectopically expressed under the cauliflower mosaic virus (CaMV) 35S promoter, and their expression was confirmed by quantitative real-time PCR (qRT-PCR) analysis. The color traits, measured by spectrophotometric colorimeter and spectral absorbance of fresh petal extracts, revealed a successful flower color modification from purple to reddish. Red pigmentation was also observed in whole plants. LC-DAD-MS and HPLC analyses confirmed that the additional accumulated pigments were betacyanins—mainly betanin (betanidin 5-*O*-glucoside) and, to a lesser extent, isobetanin (isobetanidin 5-*O*-glucoside). The five endogenous anthocyanins in torenia flower petals were also detected.

**Conclusions:**

This study demonstrates the possibility of foreign betacyanin accumulation in addition to native pigments in torenia, a popular garden bedding plant. To our knowledge, this is the first report presenting engineered expression of betalain pigments in the family Linderniaceae. Genetic engineering of betalains would be valuable in increasing the flower color variation in future breeding programs for torenia.

**Supplementary Information:**

The online version contains supplementary material available at 10.1186/s12870-024-05284-1.

## Background

The major plant pigments responsible for coloration are anthocyanins, carotenoids, and betalains [1–3]. Among them, the former two are most widespread in the plant kingdom, and their biosynthetic pathways and related enzymes are well characterized. The latter betalains constitute a different class of pigments, which exist only in plants of the order Caryophyllales. Betalains are nitrogen-containing pigments with a core structure of betalamic acid. They can be classified into two major types, red-violet betacyanins and yellow-orange betaxanthins, based on their structural characteristics and light-absorption properties [4, 5]. The betalain-biosynthetic pathway has recently been characterized, and most genes responsible for betalain biosynthesis have been identified over the past 10 years [5].

Genetic engineering is the most promising approach to drastically changing flower color that cannot be achieved by conventional breeding. For example, blue-hued flowers were engineered to accumulate delphinidin-based anthocyanins, carnation [6], rose [7], and chrysanthemum [8]. Decades ago, transgenic carnation and rose were introduced into the general markets after successfully clearing the environmental biosafety assessment of the Cartagena Protocol [9]. More recently, blue-hued Phalaenopsis, developed by a collaboration between Prof. Mii (Chiba University) and Ishihara Sangyo Kaisha Ltd., has also been commercialized [10]. Thus, producing flowers with novel colors by genetic engineering has been attempted in many ornamental plants [11–13]. Anthocyanins, a class of flavonoids, are the major plant pigments; they accumulate in many parts, including flowers, fruits, stems, roots, and leaves. They contribute to a wide range of colors, from red–orange to blue, and are considered the best targets for engineering coloration. Thus, in addition to the blue mentioned above, producing red and orange coloration is possible by suitably designing the anthocyanin components in plants. Other classes of flavonoids, like aurones and chalcones, are also targeted for the engineering of yellow-colored flowers in several plant species, including torenia [14] morning glory [15], and African violets [16]. Previously, we successfully produced pelargonidin-based red-flowered tobacco plants by combining the suppression of endogenous genes by RNAi and the overexpression of a foreign gene involved in flavonoid biosynthesis [17]. Carotenoid engineering can also be applied to improve certain crop traits [18], as demonstrated by the recent novel strategy of genome-editing targeting the *Orange* (*Or*) gene to induce β-carotene accumulation in rice [19]. Genome-editing targeting the carotenoid cleavage dioxygenase gene (*CCD4*) has also been applied to change flower color from cream or orange in morning glory [20] and *Brassica napus* [21].

Only a few studies have attempted engineering the betalain-biosynthetic pathway, compared to the many that targeted flavonoids and carotenoids. Betalains were successfully engineered in model plants, such as *Arabidopsis* and tobacco, and in crop plants, such as tomato, potato, eggplant, petunia, lisianthus, and gentian [22–27]. In these reports, betacyanin and betaxanthin were introduced, and the obtained transgenic plants exhibited a changed color, such as red, purple, and yellow, by the ectopic accumulation of betalain pigments. More recently, a versatile expression system for betalain engineering, called *RUBY*, has been developed in *Arabidopsis* and rice [28]. This system can express a single open reading frame of 2A peptide-linked betalain biosynthesis genes using a single promoter and can be used for gene expression monitoring and plant transformation. For example, *RUBY* was utilized for betalain engineering in various crops, including carrot [29], cotton [30], cannabis [31], soybean [32], and poplar [33]. The *RUBY* reporter is also used as a biotechnological tool; for example, for efficient haploid identification in maize and tomatoes [34] and genome editing in soybeans as a visual indicator [35].

However, despite many successful examples, the genetic engineering of betalains in ornamental plants is still limited. This study examined the possibility of betalain engineering in the popular ornamental flower plant, torenia. Torenia is the common name for several species in the genus, including *T. fournieri*, *T. concolor*, *T. asiatica*, and *T. hybrida* (*T. fournieri* × *T. concolor*) [36]. Torenia belongs to the family Linderniaceae, separated from the traditionally defined Scrophulariaceae. Due to its easy handling and the availability of an efficient transformation system, this plant has been used as a model ornamental plant in several studies [37–40]. Blue is the wild-type flower color in *T. fournieri* and *T. concolor*, and other colors, such as pink, white, and purple, have also been bred by conventional breeding [41]. Additionally, flower color in torenia plants has been modified by regulating the flavonoid-biosynthetic pathway [42–44]. We have also previously demonstrated efficient flower color modification in torenia by targeting the flavanone 3-hydroxylase (*F3H*) gene with the CRISPR/Cas9 system [45]. Thus, a biotechnological approach is promising for increasing flower color variation in torenia but betalain engineering has not yet been achieved in torenia plants.

In this study, we attempted to engineer the betalain pigments in the purple-flowered torenia cultivar “Crown Violet”, as depicted in Supplementary Fig. S1. *Beta vulgaris* cytochrome P450 (BvCYP76AD1) [46], is a P450-type protein that catalyzes the first two steps in betalain biosynthesis, i.e., tyrosine to L-DOPA and L-DOPA to *cyclo*-DOPA. In *Mirabilis jalapa*, *cyclo*-DOPA is glucosylated by *cyclo*-DOPA-5-*O*-glucosyltransferase (MjcDOPA5GT) [47] to form *cyclo*-DOPA 5-*O*-glucoside, and DOPA 4,5-dioxygenase (MjDOD) [48] converts L-DOPA to betalamic acid. Betacyanins are formed by the spontaneous conjugation of *cyclo*-DOPA 5-*O*-glucoside and betalamic acid. The results clearly indicated that introducing three genes—*BvCYP76AD1* from *B. vulgaris*, *MjDOD* and *MjcDOOA5GT* from *M. jalap*a—involved in betalain biosynthesis induced the ectopic accumulation of two betacyanins, betanin and isobetanin, and modified the flower color from purple to reddish–purple, as expected. We also discuss the future molecular breeding of torenia for flower color alteration by betalain engineering.

## Methods

### Plant materials and production of torenia transformants

*Torenia fournieri* “Crown Violet” was aseptically propagated on Murashige and Skoog (MS) medium supplemented with 3% (w/v) sucrose and 0.2% (w/v) gellan gum. This cultivar is a clonal line provided by Dr. Ryutaro Aida (National Institute of Floricultural Science, Japan) derived from the Crown series. *Agrobacterium*-mediated transformation was performed as described previously [49].

### Construction of a binary vector for torenia transformation

Three genes, *BvCYP76AD1* [46], accession no. KU644144, *MjcDOPA5GT* [47], accession no. AB182643, and *MjDOD* [48], accession no. AB435372, involved in betalain biosynthesis (Fig. S1), were cloned under the control of the *CaMV35S* promoter and Arabidopsis heat shock protein terminator [50] in tandem into the binary vector *pSKAN221-HT* [51], thus obtaining pSKAN-35SpBvCYP76AD1&MjcDOPA5GT&MjDOD-HT. The binary vector comprised an *NPTII* expression cassette for the selection of transgenic plants based on kanamycin resistance. The binary vector was transformed into *Agrobacterium tumefaciens* strain EHA101 by electroporation and used for torenia transformation.

### qRT-PCR analysis of foreign genes in transgenic torenia plants

Expression levels of introduced foreign genes were analyzed as described previously [25]. Potted plants, after acclimatization for two to three months, were used for the analysis. Flowers were collected just after anthesis, and petals were separated from the flowers. The leaves were sampled at the same time points as the flowers. Individual flowers or leaves were stored in 2 mL screw cap tubes at − 80 °C until use. Samples from one flower or one leaf were used per analysis. Total RNA was extracted from flower petals and leaves, and cDNA was synthesized using the ReverTra Ace® qPCR RT Master Mix with gDNA Remover (Toyobo, Osaka, Japan) according to the manufacturer’s instructions. The QuantStudio Real-Time PCR system (Applied Biosystems Japan) was used for qRT-PCR analysis. The qRT-PCR primer sets for *BvCYP76AD1*, *MjDOD*, *MjcDOPA5GT*, and *TfACT3* (reference gene) are listed in the Supplemental Table S1. A melting curve analysis was performed to verify the specificity and identity of the qRT-PCR products. The expression levels of foreign genes were normalized against the *TfACT3* expression level and expressed as relative values.

### Measurement of petal colors

To quantify the flower color phenotypes of the betacyanin-accumulating transgenic plants and wild-type (WT) plants, the colorimetric values L*, a*, and b*, the chroma (C*) value, and hue angle of the adaxial surface of the lip and tube area of fresh petals were measured using the benchtop CM-3600 A spectrophotometer (Konica Minolta, Tokyo, Japan). In this analysis, four to five independent flowers per line—transgenic and WT—were used.

### LC-DAD-MS analysis

Flower petals were collected from plants, immediately frozen in liquid nitrogen, and stored at − 80 °C until analysis. The samples were prepared as described previously [25]. Betacyanins and anthocyanins were analyzed by liquid chromatography-diode array detection-quadrupole time-of-flight mass spectrometry apparatus (LC-DAD-TOFMS; Agilent Technologies). Samples were separated using a C30 column (4.6 mm × 250 mm; Numura Chemical Co., Seto, Japan) using a binary mobile phase composed of 0.1% (v/v) formic acid (A) and methanol containing 0.1% (v/v) formic acid (B) at 40 °C. The gradient was as follows: 0 min, 40% B; 20 min, 60% B; and 25 min, 60% B. The flow rate was 0.3 ml/min. LC-DAD chromatograms were acquired at 535 nm, and the UV-vis spectra were recorded at 400–600 nm.

### Analysis of pigment compositions and concentrations in petals and leaves

To determine pigment compositions and concentrations, the petals were cut into lip and tube immediately after anthesis of flowers. The petal lips, petal tubes, and leaves were immediately frozen in liquid nitrogen and stored at − 80 °C until further use. The samples (about 100 mg) were then powdered in a 2 mL microtube with 0.2 g ceramic beads (2 mm i.d.) using a bead-homogenizer MS-100 (TOMY, Tokyo, Japan) at 5,500 rpm for 20 s. Powdered samples were further homogenized with 3 volumes of 80% (v/v) methanol containing 1% (v/v) formic acid at 5,500 rpm for 20 s. The resulting suspensions were centrifuged at 13,200 rpm for 5 min at 4 °C. Subsequently, 150 µL aliquots were cleared using the Cosmospin Filter H (0.45 μm; Nacalai Tesque Inc., Kyoto, Japan). Combined supernatants were analyzed using the HPLC system comprising an Agilent 1200 series (Agilent Technologies, CA, USA). The InertSustain C18 column (250 × 4.6 mm internal diameter, 5 μm; GL Sciences Inc., Tokyo, Japan) was used with a column temperature of 40 °C. Mobile phases were applied at 0.6 mL/min and comprised solvents A (water/formic acid, 97:3, v/v) and B (water/acetonitrile/formic acid, 67:30:3, v/v/v): 25% B for 15 min, followed by a linear increase from 25% B to 50% B over 50 min, and 50% B for 1 min. The injection volume was 10 µL. Photodiode array detector (PDA) was used at 540 nm.

For the analysis of the absorbance spectra, petal lips, petal tubes, and leaves were powdered as described above and extracted with 50% methanol containing 1% formic acid overnight at 4 °C with gentle shaking. After centrifugation at 15,000 rpm for 5 min at 4 °C, the supernatants were recovered and diluted suitably with the same extraction solution. The absorbance spectra were measured using a visible spectrophotometer (ASV-S3; AS ONE, Osaka, Japan).

### Statistical analysis

Colorimetric values and betacyanin contents, determined by spectrophotometer and HPLC analysis, respectively, were analyzed by applying Dunnett’s multiple comparison test to evaluate the significant differences between the control and transgenic plants.

## Results

### Production of transgenic torenia plants

**Fig. 1:**
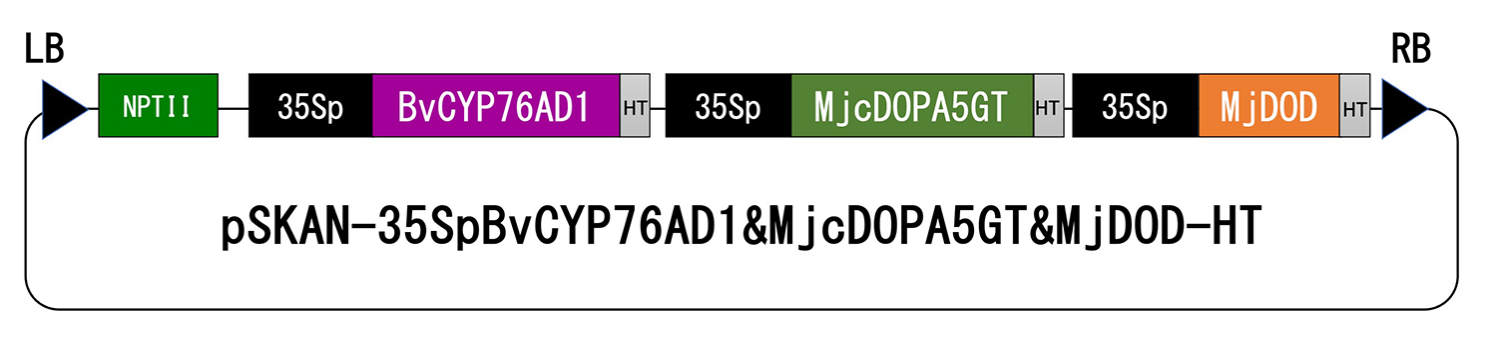
Schematic of the binary vector for the ectopic production of betacyanins. LB, left border of T-DNA; *NPTII*, expression cassette for the selection marker neomycin phosphotransferase II; 35Sp, Cauliflower mosaic virus 35 S promoter; *BvCYP76AD1*, *Beta vulgaris* cytochrome P450 (accession no. KU644144); *MjcDOPA5GT*, *Mirabilis jalapa* cyclo-DOPA-5-*O*-glucosyltransferase (accession no. AB182643); *MjDOD*, *M. jalapa* DOPA 4,5-dioxygenase (accession no. AB435372); HT, terminator of heat shock protein 18.2 of *A. thaliana*; RB, right border of T-DNA

We constructed a binary vector to induce the constitutive expression of betalain (betacyanin)-biosynthetic genes under the control of the *CaMV35S* promoter (Fig. 1). The process of generating transgenic plants progressed without difficulty, and many red-colored calli appeared during selection on the kanamycin-containing regeneration medium. The regenerated plantlets from the red calli were also entirely red, including stems, leaves, and roots (Supplementary Fig. S2A), and were maintained by in vitro culture (Supplementary Fig. S2B). We then selected three transgenic lines displaying a reddish color and acclimatized and cultivated them in a closed glasshouse to facilitate flowering.

### Confirmation of gene expression in torenia plants by qRT-PCR analysis

qRT-PCR analysis was performed in two transgenic torenia lines to determine the expression levels of the transgenes. All three genes, *BvCYP76AD1*, *MjDOD*, and *MjcDOPA5GT*, were expressed in petals and leaves, and similar expression levels were found in the two lines (Supplementary Fig. S3). No expression was detected in WT plants.

### Phenotype of transgenic torenia plants

**Fig. 2:**
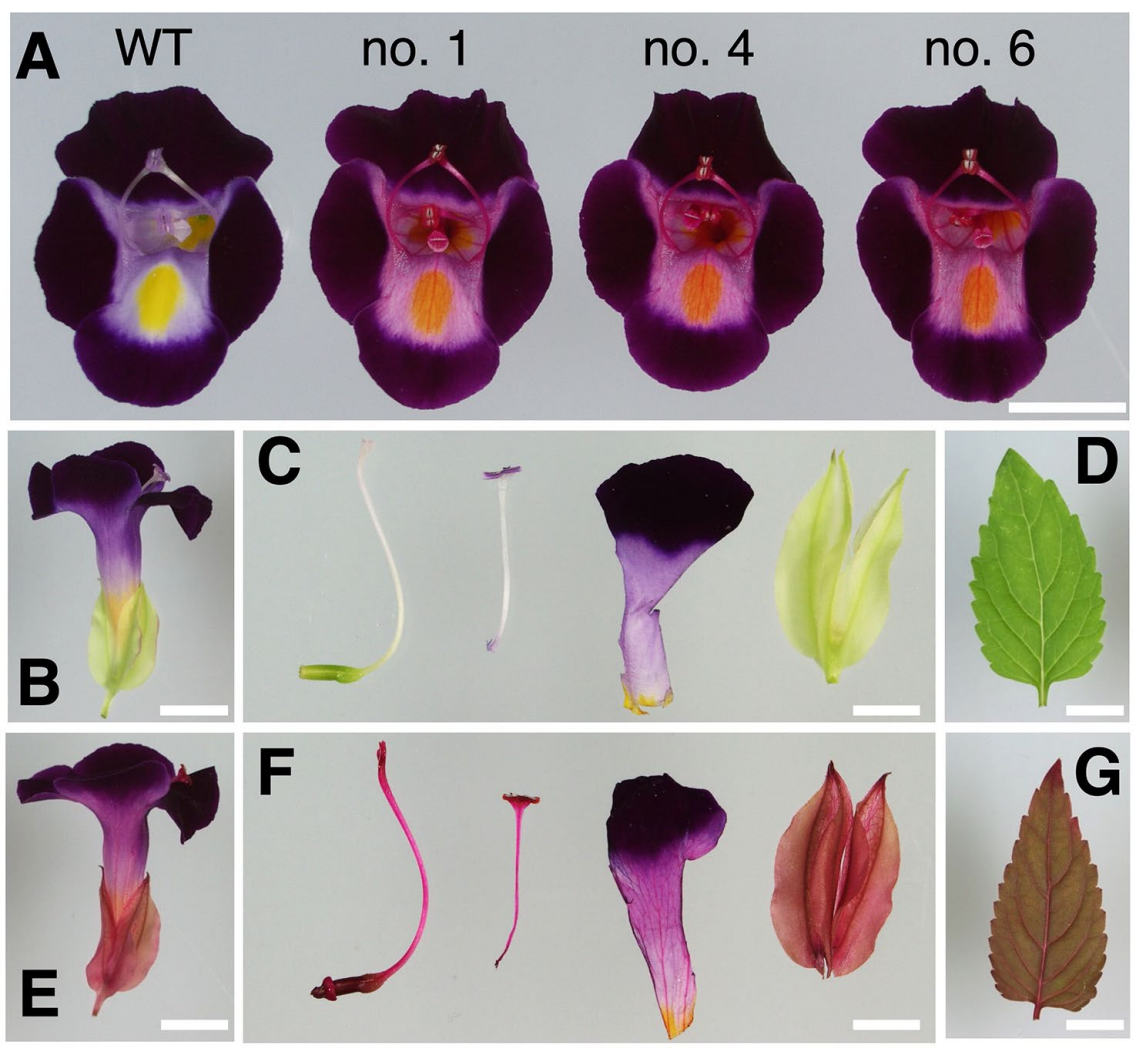
Pictures of WT and transgenic torenia plants. (**A**) From left to right, top view of WT, Bc-no. 1, Bc-no. 4, and Bc-no. 6 flowers. (**B–D**) WT flower parts and leaf. (**E–G**) Bc-no. 1 flower parts and leaf. (**B, E**) Lateral view of flowers. (**C, F**) Separated flower organs. From left to right: pistil, stamen, petal, and sepal. (**D, G**) Leaves. Scale bars: 1 cm

Typical pictures of three lines of transgenic and WT plants are presented in Fig. 2 and Supplementary Fig. S4. The three transgenic torenia lines displayed a remarkable color change to reddish–purple flowers from the WT purple (Fig. 2A, Supplementary Fig. S4). Lateral view of flowers also indicated reddish pigmentation (Fig. 2E) compared with the WT (Fig. 2B). Flower organs, including pistil, stamen, petal, and sepal, had also reddish pigmentation (Fig. 2F) compared with the WT (Fig. 2C). Transgenic torenia plants also had reddish leaves, stems, and roots (Fig. 2G, Supplementary Fig. S4 and Fig. S2B), whereas WT plants had green leaves and stems (Fig. 2D, Supplementary Fig. S4, and Fig. S2B). Because these three lines displayed a similar appearance, we selected two lines (named Bc-no. 1 and Bc-no. 4) and subjected them to further analysis.

### Evaluation of flower petal color of transgenic torenia

To evaluate the color difference between WT and transgenic lines, we analyzed the colorimetric traits of the adaxial surface of petals by spectrophotometry. As “Crown Violet” has dark purple petal lips and light purple petal tubes (Fig. 2A), we measured color traits in these two parts (Supplementary Fig. S5A). CIE L*a*b* values, in which L* indicates lightness, a* is the red/green coordinate, and b* is the yellow/blue coordinate, are indicated in Table 1. The a* and Chroma values, indicating red color intensity and brightness, of Bc-no. 1 and Bc-no. 4 were significantly higher than those of WT plants in the petal lip and tube. L* values and hue (*h* °) were also significantly different between WT and transgenic lines in the petal tube, but the difference was not significant in the petal lip except for L* value of Bc-no. 1. None of the parameters was significantly different between Bc-no.1 and Bc-no. 4. These results confirmed the visible differences in flower color, especially in the petal tube (Fig. 2), between the WT and transgenic lines.

**Table 1:** Spectrophotometer-based color measurement of the surface of fresh petals

		L*	a*	b*	Chroma	Hue (h°)
WT	Lip	1.6 ± 0.7	2.3 ± 0.7	-0.3 ± 0.4	2.4 ± 0.7	351.8 ± 10.6
Bx-no. 1		3.4 ± 1.5*	3.6 ± 0.5*	-0.6 ± 0.7	3.7 ± 0.5*	350.7 ± 9.9
Bx-no. 4		2.1 ± 0.7	3.6 ± 0.9*	-0.5 ± 0.6	3.6 ± 1.0*	353.8 ± 6.3
WT	Tube	64.3 ± 1.9	4.9 ± 1.3	-14.8 ± 4.5	15.6 ± 4.7	288.7 ± 0.8
Bx-no. 1		49.2 ± 2.0**	27.4 ± 1.1**	-15.1 ± 3.0	31.4 ± 1.8**	331.3 ± 4.7**
Bx-no. 4		50.4 ± 0.7**	26.9 ± 0.6**	-14.4 ± 1.5	30.5 ± 1.1**	331.9 ± 2.3**

L*, a*, and b* color values at the surface of fresh petals were measured with spectrophotometer

L* indicates lightness, a* is the red/green coordinate, and b* is the the yellow/blue coordinate

Asterisks indicate significant differences compared with WT as demonstrated by Dunnett’s multiple comparison test (*, *P* < 0.05; **, *P* < 0.01)

### Pigment analysis of transgenic torenia petals and leaves

**Fig. 3:**
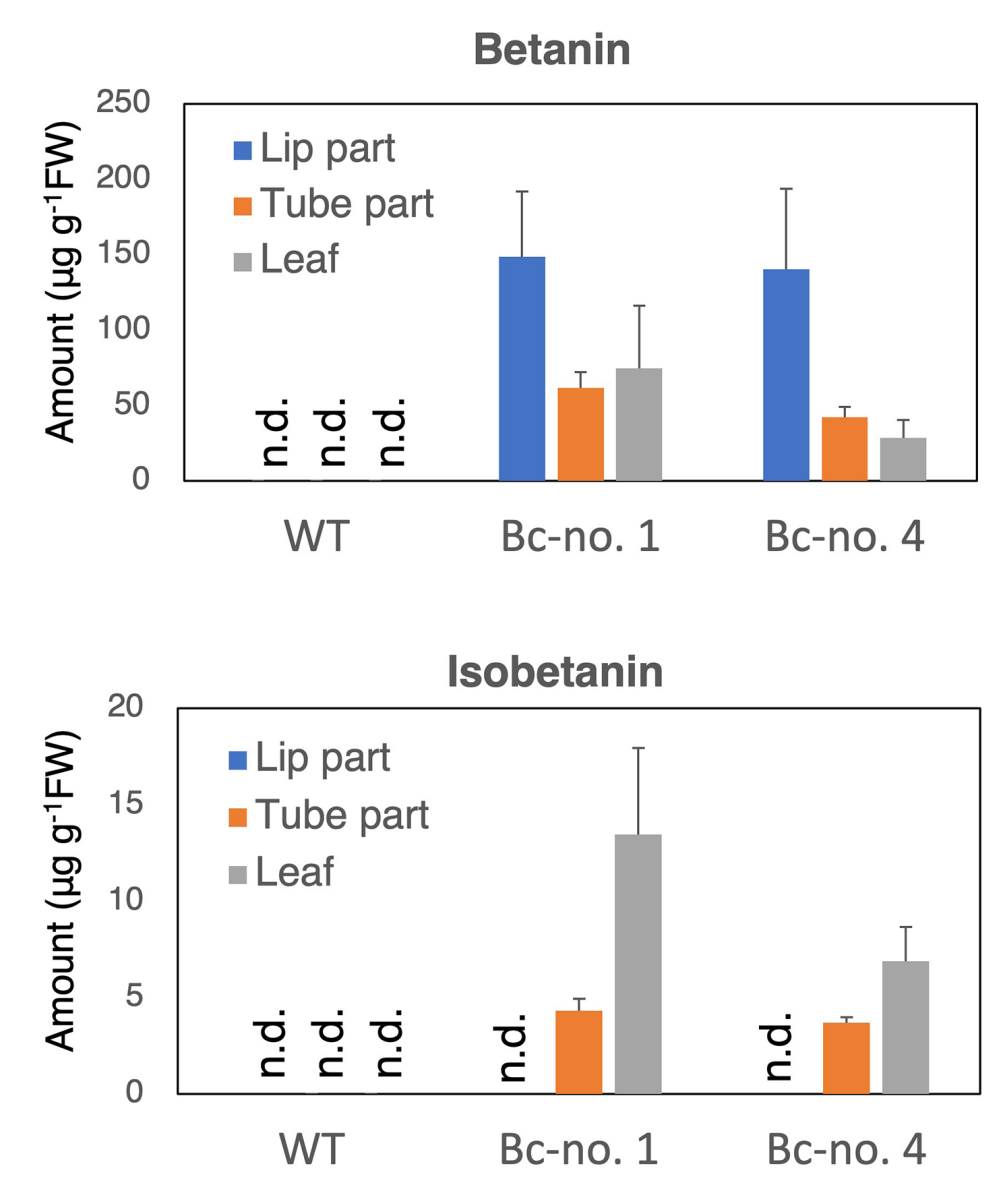
Betacyanin content in WT and transgenic torenia petals and leaves. Betanin and isobetanin concentrations were determined by HPLC analysis. Error bars represent the standard deviations of the means of five samples. n.d., not detected

**Fig. 4:**
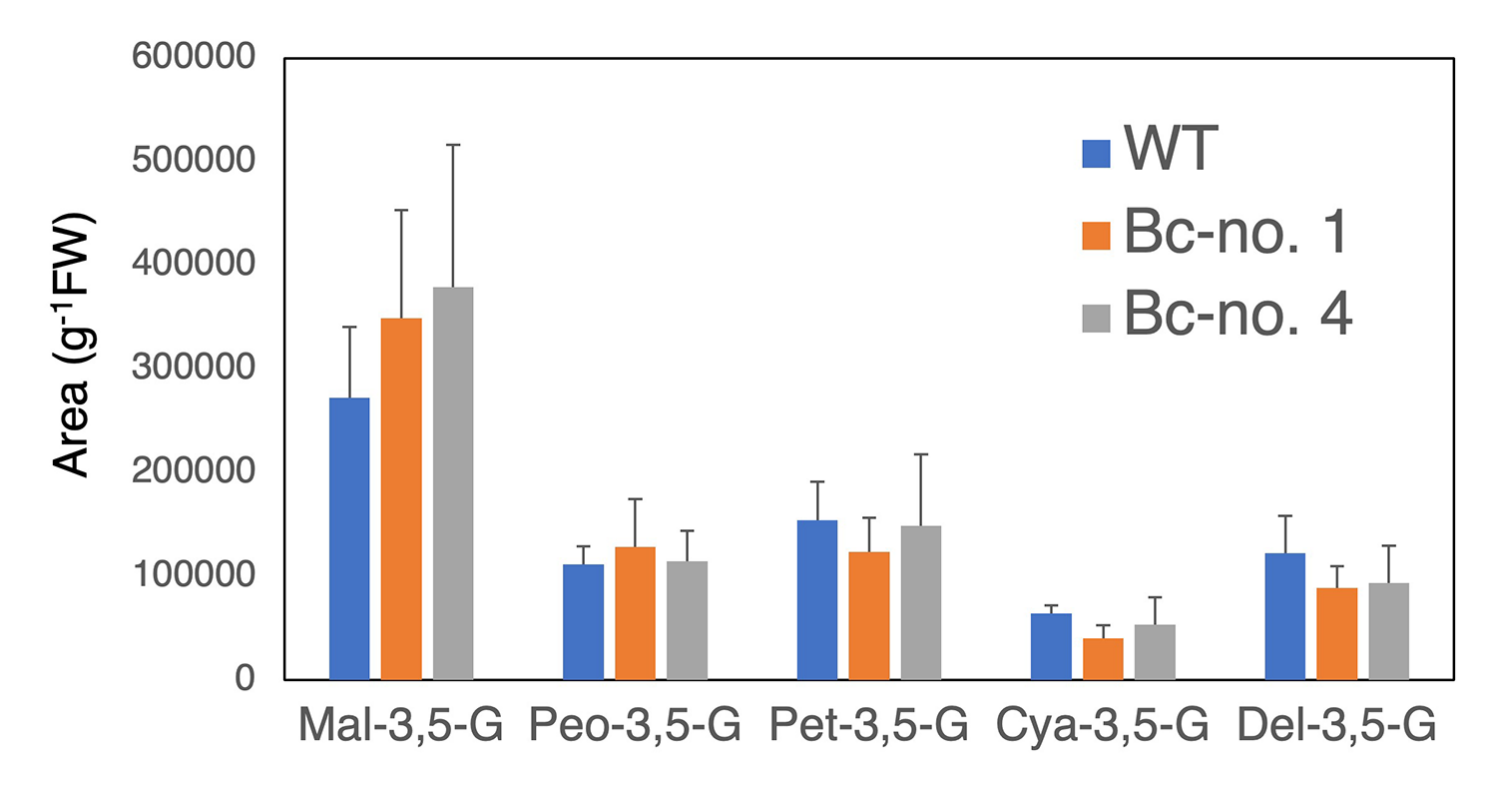
Anthocyanin content in WT and transgenic torenia petal tubes. Peak areas of five anthocyanins determined by HPLC analysis per gram fresh weight. Error bars represent the standard deviations of the means of five samples

**Fig. 5:**
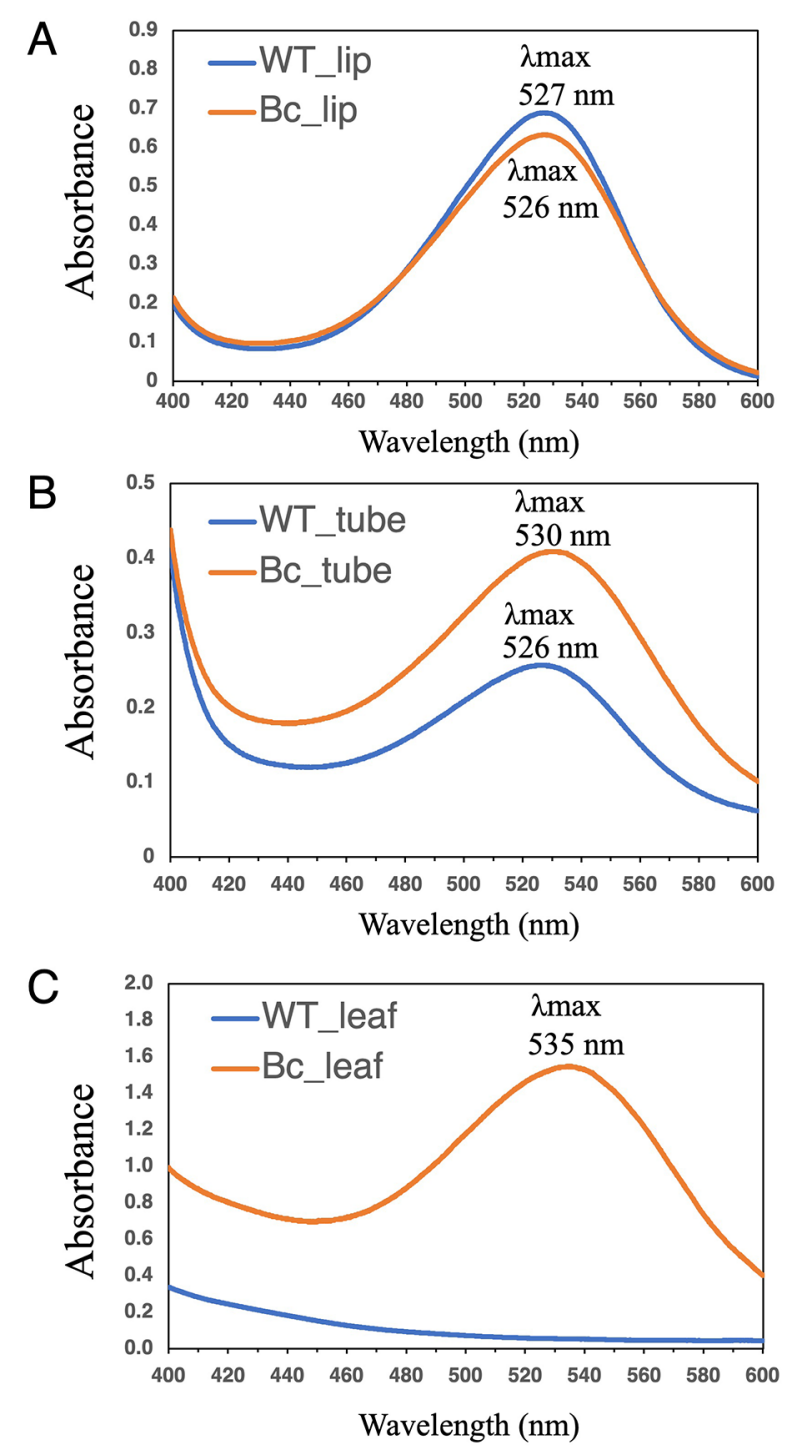
Absorbance spectra of WT and Bc-no. 1 petals and leaves. Absorbance spectra of petal lip (**A**), petal tube (**B**), and leaf (**C**) extracts

To identify the accumulated pigments, LC-DAD-MS analysis was first performed using whole petals (Supplementary Fig. S6). Five anthocyanins, delphinidin 3,5-diglucoside (Del-3,5-G), cyanidin 3,5-diglucoside (Cya-3,5-G), petunidin 3,5-diglucoside (Peo-3,5-G), peonidin 3,5-diglucoside (Pet-3,5-G), and malvidin 3,5-diglucoside (Mal-3,5-G), were detected in this cultivar. An additional peak 1 was also detected in transgenic torenia petal. The m/z of peak 1 was 551.151, corresponding to two betacyanins, betanin (betanidin 5-*O*-glucoside) and isobetanin (isobetanidin 5-*O*-glucoside). The absorbance λ max of the additional peak 1 was 535 nm, corresponding to that of betanin and isobetanin. Next, to measure the concentrations of each of the anthocyanins and betalains accumulated in petal lips, petal tubes, and leaves, we performed HPLC analysis using the optimized method to separate these compounds clearly. The petal lip and tube parts used are indicated in Figure S5B. Betanin and isobetanin were detected in petal tubes and leaves, but isobetanin was not detected in petal lips (Figs. 3 and 4). Betanin amounts were approximately 4–13 times higher than isobetanin amounts in petal tubes and leaves (Fig. 3). The five anthocyanins are displayed as peak areas of HPLC chromatograms (Fig. 4). The total peak areas of these five anthocyanins were similar between genotypes: 724,884 ± 71,252, 730,414 ± 95,867, and 790,484 ± 120,694 per gram fresh weight for WT, Bc-no. 1, and Bc-no. 4, respectively. No significant difference was observed in the accumulated anthocyanin content between WT and transgenic lines. Absorbance spectra of petal lip and tube extracts are presented in Fig. 5. Absorbance λ max shifted from 526 nm (WT) to 530 nm (Bc-no.1) in the petal tube, but little difference was observed in the petal lip. Absorbance λ max of Bc-no. 1 leaf extract was 535 nm, corresponding to that of betanin, whereas no peak was detected in WT leaves.

## Discussion

Evolutionarily, betalains are only synthesized and accumulated by Caryophyllales [52–54]. Betalains are substitute pigments for anthocyanins and play important roles not only in coloration but also in plant defenses [55]. Although functionally similar, betalains and anthocyanins are mutually exclusive and never occur together in the same plant in nature. However, using modern biotechnology and our knowledge of the betalain-biosynthetic pathway and the related genes, we can genetically engineer non-Caryophyllales plant species to produce betalains [5]. Betalains are relatively stable over a broad pH range of 3–7 [56]. Although betalains are degraded by increasing temperature, betacyanins are more stable than betaxanthins, at room temperature [57] and upon heating [58]; therefore, betacyanins can be used as natural food colorants. Furthermore, betalains are biologically active compounds. They are antiinflammatory, anticancer, antilipidaemic, antimicrobial, and free radical-scavengers [59–61], thus good candidates for the development of functional foods. So far, more than ten plant species belonging to the families of Solanaceae, Brassicaceae, Gentianaceae, Cannabaceae, Malvaceae, Apiaceae, Fabaceae, and Poaceae were genetically engineered to accumulate betalains in various organs, including leaves, stems, flowers, tubers, and fruits [22–27, 29–32, 34, 35, 62]. Several ornamental plants, such as petunia [23], lisianthus [24], and gentian [25], have also been reported to change flower color after being genetically transformed for betalain synthesis and accumulation. Betalains are more stable than anthocyanins over a wide pH range [56] and give vivid red and yellow coloration, as determined by fluorescence [63]. Therefore, the engineered expression of betalains in plant species originally having red and yellow-colored flowers can bring novel red and yellow hues. The blue color is an exclusive feature of anthocyanins, but torenia has naturally blue-colored flowers. Thus, we focused on torenia, a popular summer bedding plant grown worldwide [36], and attempted to further beautify the flower coloration by betalain engineering. Flowers of *T. fournieri* are edible and used as salad [64]. Therefore, the successful accumulation of betalains in torenia flowers would enhance the functional component of this food. Our finding would also facilitate the engineering of novel flower colors by modulating betalain accumulation in other ornamental flowers.

Absorbance maxima (λ max) for individual anthocyanins, such as Mal-3,5-G, Peo-3,5-G, Cya-3,5-G, Pet-3,5-G, and Del-3,5-G are reported around 511–525 nm [65, 66], and those of betanin and isobetanin are 535 nm [67]. Therefore, the shift from 526 nm (WT) to 530 nm (Bc) in petal tube confirms the presence of betalains in the transgenic line (Fig. 5B). This result was confirmed by spectrophotometric colorimetry using fresh flower petals (Table 1), where petal tubes rather than petal lips displayed significantly different values in trangenic lines compared to WT torenia flowers. We hypothesize that, as petal lips contain higher anthocyanin levels, the accumulation of additional betacyanins have only a small effect on the flower coloration, as measured spectrophotometrically or visually observed. Regarding petal tubes, the original anthocyanin amounts are small, and the addition of novel betacyanins is visible and can be clearly detected experimentally. Therefore, using host plants with white-colored flowers that do not accumulate colored pigments would result in clear betalain coloration. Many white-flowered torenia cultivars are available, such as “Kauai white” and “Little kiss white” [41]. We also identified a white-flowered “Crown White” as an *F3H* mutant [49]. Genome-editing technology can be used to produce white-flowered torenia plants efficiently [45], and using them as host plants would be expected to give promising results in flower color modification by transformation with betalain-biosynthetic pathway genes. Additionally, our transgenic torenia lines had red pigmentation in the whole plant, including leaves, stems, and roots, but no deleterious effects were observed. In contrast, deleterious effects due to the constitutive expression and accumulation of betaxanthin were reported in gentian [25]. Hence, inducing petal-specific expression, as demonstrated in the production of yellow-flowered gentians, might be a better solution [25]. Several petal-specific promoters of MADS box genes involved in floral organ identity and flavonoid-biosynthetic genes for pigmentation have been identified in torenia [68]. Further studies using these promoters would help specifically change flower petal color.

We demonstrated that anthocyanin components and contents were not affected by betacyanin production in petal lips (Fig. 4). Although a detailed analysis is necessary, we hypothesize that this observation could be due to two biosynthetic pathways operating independently of each other, therefore not competing for substrates (phenylalanine for anthocyanins and tyrosine for betalains). Betacyanin biosynthesis can be enhanced by introducing arogenate dehydrogenase (ADHα), which has a relaxed sensitivity to the negative feedback inhibition by tyrosine [69]. More recently, L-DOPA 4,5-dioxygenase was also identified as a critical rate-limiting step of the betalain-biosynthetic pathway [70]. Thus, engineering of these genes would boost betalain accumulation in higher plants. Coaccumulation of anthocyanins and betalains can potentially offer greater choice for desired combinations of flower colors in genetically engineering ornamental plants.

## Conclusions

In this study, we genetically engineered torenia plants to synthesize and accumulate betalain (betacyanin). Coaccumulation of anthocyanins and betacyanins in flower petals resulted in reddish flower color as previously reported in lisianthus using the same gene set [24]. This is the first report of successful engineering of betalain pigment accumulation in the popular ornamental torenia flower. Our results would help improve the molecular breeding of various flower colors in torenia by combining these two types of pigments. Yellow betaxanthins, another class of betalains, can also be introduced to create vivid yellow-colored torenia in the future.

### Electronic supplementary material

Below is the link to the electronic supplementary material.

Supplementary Material 1

Supplementary Material 2

## Data Availability

Data is provided within the manuscript or supplementary information files.

## Abbreviations

CaMV: Cauliflower mosaic virus
DOPA: Dihydroxyphenylalanine
HPLC: High-performance liquid chromatography
LC-DAD-TOFMS: Liquid chromatography-diode array detection-quadrupole time-of-flight mass spectrometry apparatus
qRT-PCR: Quantitative real-time PCR
