# Supplementary material for: Flower color modification in Torenia fournieri by genetic engineering of betacyanin pigments
Source: BMC Plant Biol. 2024 Jun 27;24:614. doi: 10.1186/s12870-024-05284-1 (PMC11210153; doi:10.1186/s12870-024-05284-1)
Supplement: Supplementary file 1 — Supplementary Material 1 [file 12870_2024_5284_MOESM1_ESM.pdf]

Table S1 Primers used in this study

Target gene	Primer Name	Sequence	Note
**For vector construction**			
BvCYP76AD1	BvCYP76AD1start	TCTAGAATGGATCATGCAACATTAGCAATG	Forward primer
	BvCYP76AD1stop	CTCGAGTCAATACCTAGGTATTGGAATAAG	Reverse primer
MjDOD	MjDODstart	TCTAGATGAAAGGAACATACTATATAA	Forward primer
	MjDODstop	GTCGACTTAATCAGTTTTTGAGTGGTG	Reverse primer
MjDOPA5GT	MjDOPA5GTstart	TCTAGATGACCGCCATTAAAATGAACAC	Forward primer
	MjDOPA5GTstop	GTCGACTTATTGAAGAGAAGGTTCCAACT	Reverse primer
**For qRT-PCR**			
BvCYP76AD1	BvCYP76AD1_F	CAACGACGACTGATGATGTGCTA	Forward primer
	BvCYP76AD1_R	CTCGCCCATAGTGAGCTCATT	Reverse primer
MjDOD	MjDOD_F	TACCCTTTGCATGTGGCATTAG	Forward primer
	MjDOD_R	CAGCCCAGCTTCGATGAATAA	Reverse primer
MjDOPA5GT	MjDOPA5GT_F	TGCTAAAGACGTGGGCTCAAC	Forward primer
	MjDOPA5GT_R	GGACACATATGCGCTTGTACCA	Reverse primer
ACTIN	TfACT3_F	GTCAGCCACACAGTCCCAATC	Forward primer
	TfACT3_R	AGCGAGATCAAGACGGAGGAT	Reverse primer
