# Supplementary material for: Flower color modification in Torenia fournieri by genetic engineering of betacyanin pigments
Source: BMC Plant Biol. 2024 Jun 27;24:614. doi: 10.1186/s12870-024-05284-1 (PMC11210153; doi:10.1186/s12870-024-05284-1)
Supplement: Supplementary file 2 — Supplementary Material 2 [file 12870_2024_5284_MOESM2_ESM.pdf]

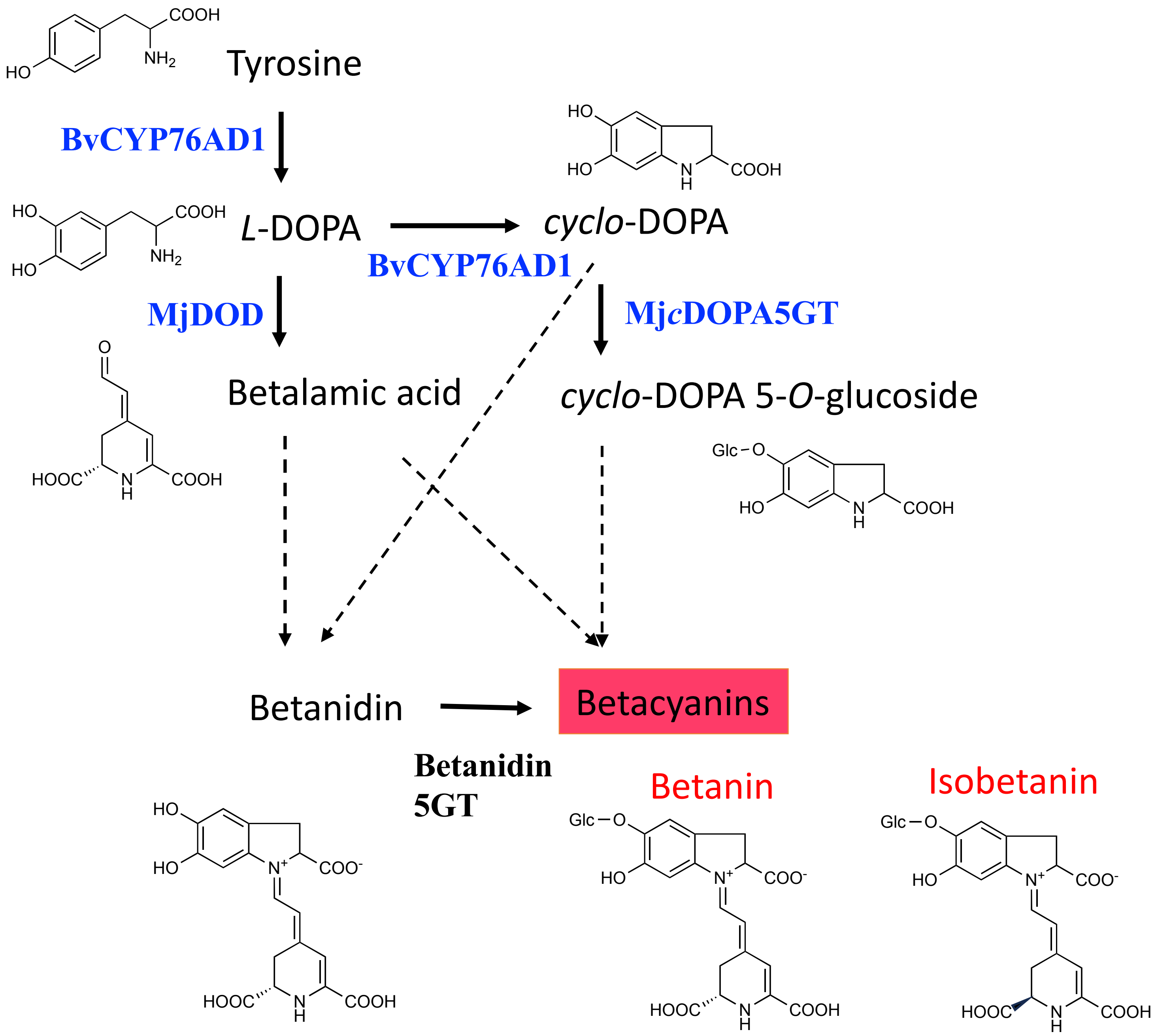

Supplementary Figures S1.
Betacyanin biosynthetic pathway.

Solid arrows and dotted arrows indicate enzymatic reactions and spontaneous reactions, respectively. Genes used for betacyanin engineering are presented in blue. Betanin also exists as its optical isomer, isobetanin.

BvCYP76AD1, *Beta vulgaris* cytochrome P450;
MjDOD, *Mirabilis jalapa* DOPA 4,5-dioxygenase;
MjcDOPA5GT, *M. jalapa* cyclo-DOPA-5-*O*-glucosyltransferase;
Betanidin 5GT, betanidin 5-*O*-glucosyltransferase.

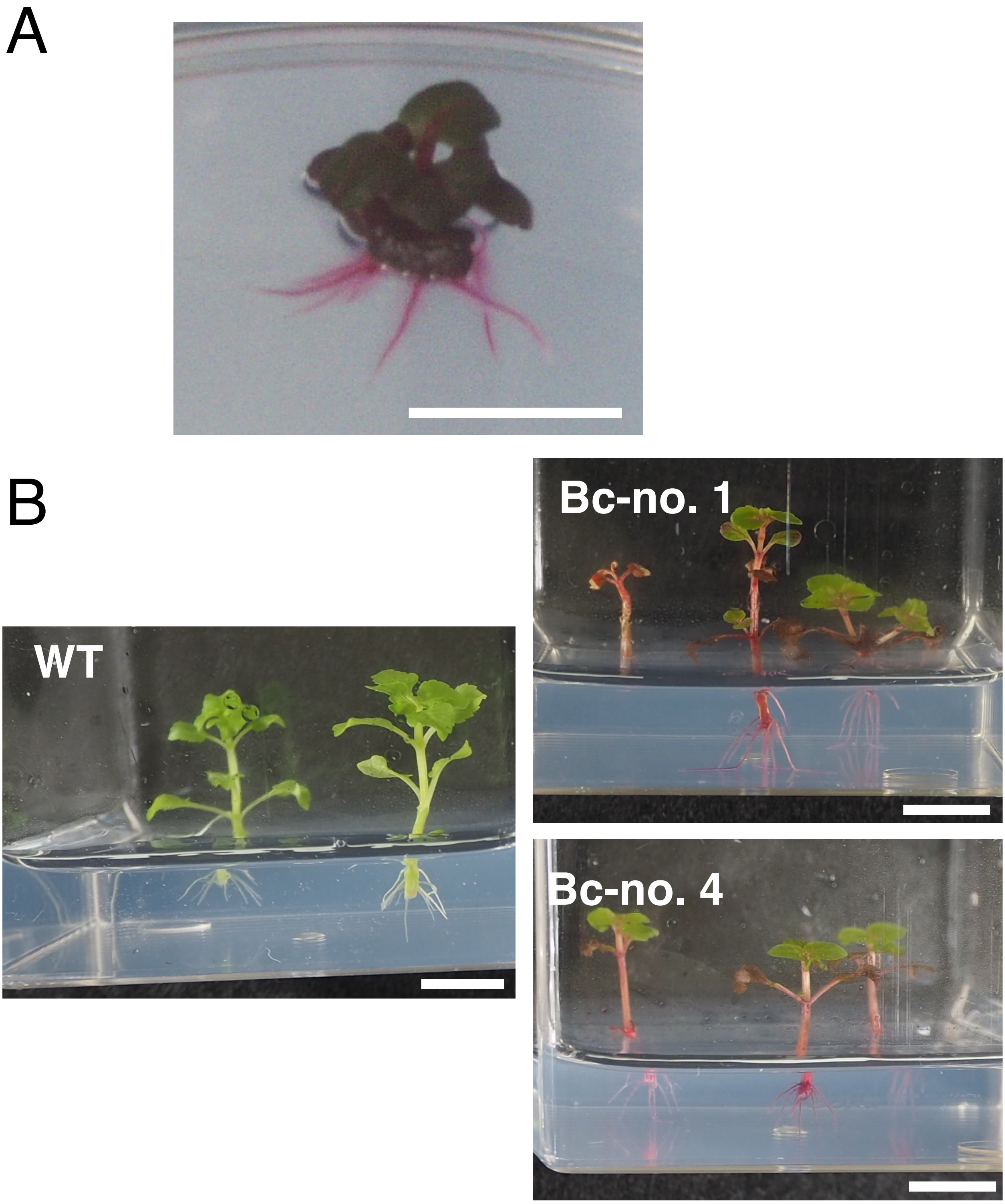

Supplementary Figure S2.
Pictures of *in vitro*-cultured torenia plantlets.

(A) *In vitro*-regenerated plantlet from red callus transformed with pSKAN-35SpBvCYP76AD1& MjcDOPA5GT&MjDOD-HT.
(B) WT and two transgenic lines (Bc-no. 1 and Bc-no. 4) propagated by in vitro culture.

Scale bars: 1 cm.

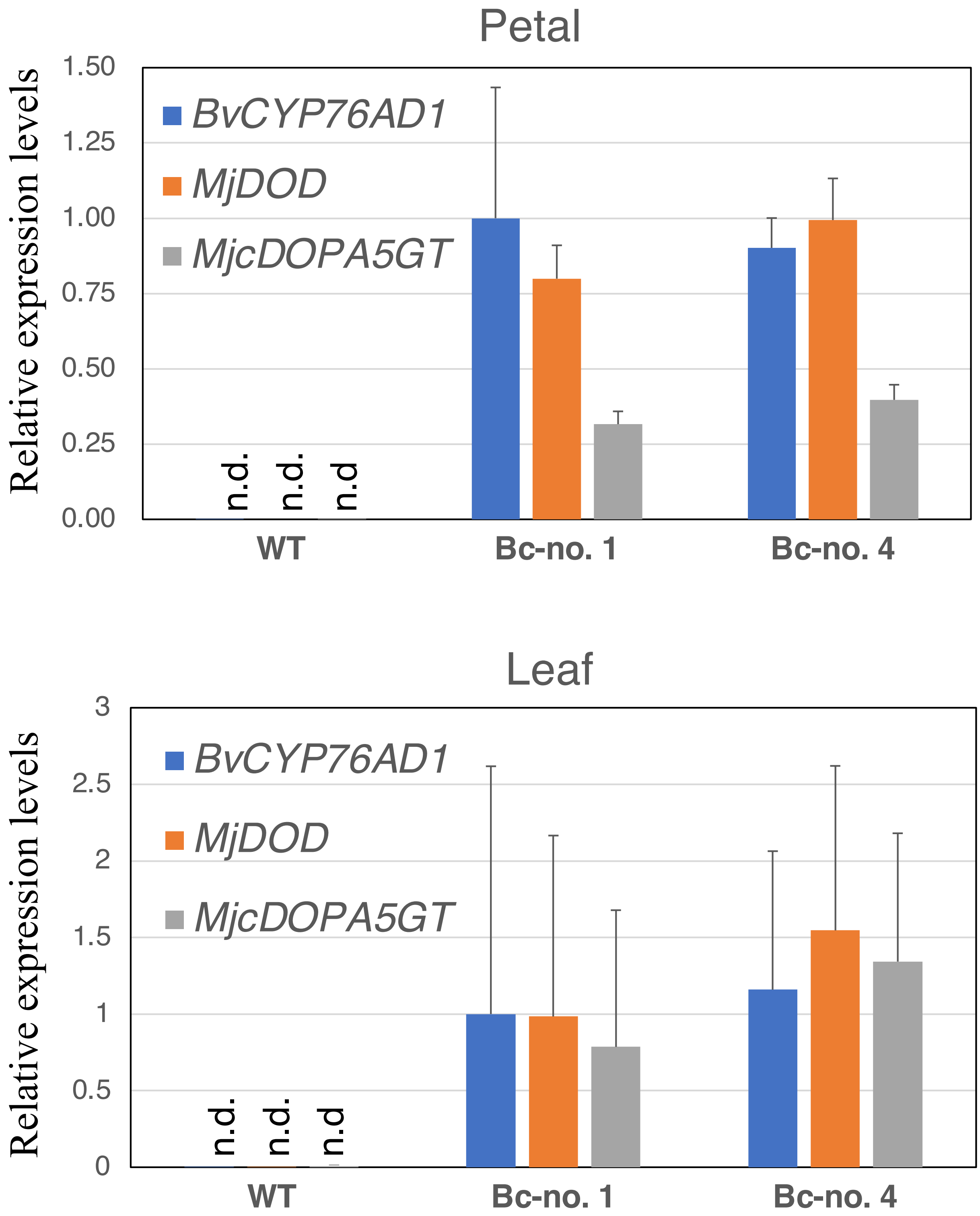

Supplementary Figure S3.
qRT-PCR analysis of introduced betalain-biosynthetic genes in transgenic torenia plants.

Relative expression levels using a torenia actin gene (*TfACT3*) as an internal control. Bc-no. 1 of *BvCYP76AD1* is set as 1.
Petals from five different flowers and leaves were used for the analysis.
Error bars indicate standard deviation. n.d., not detected.

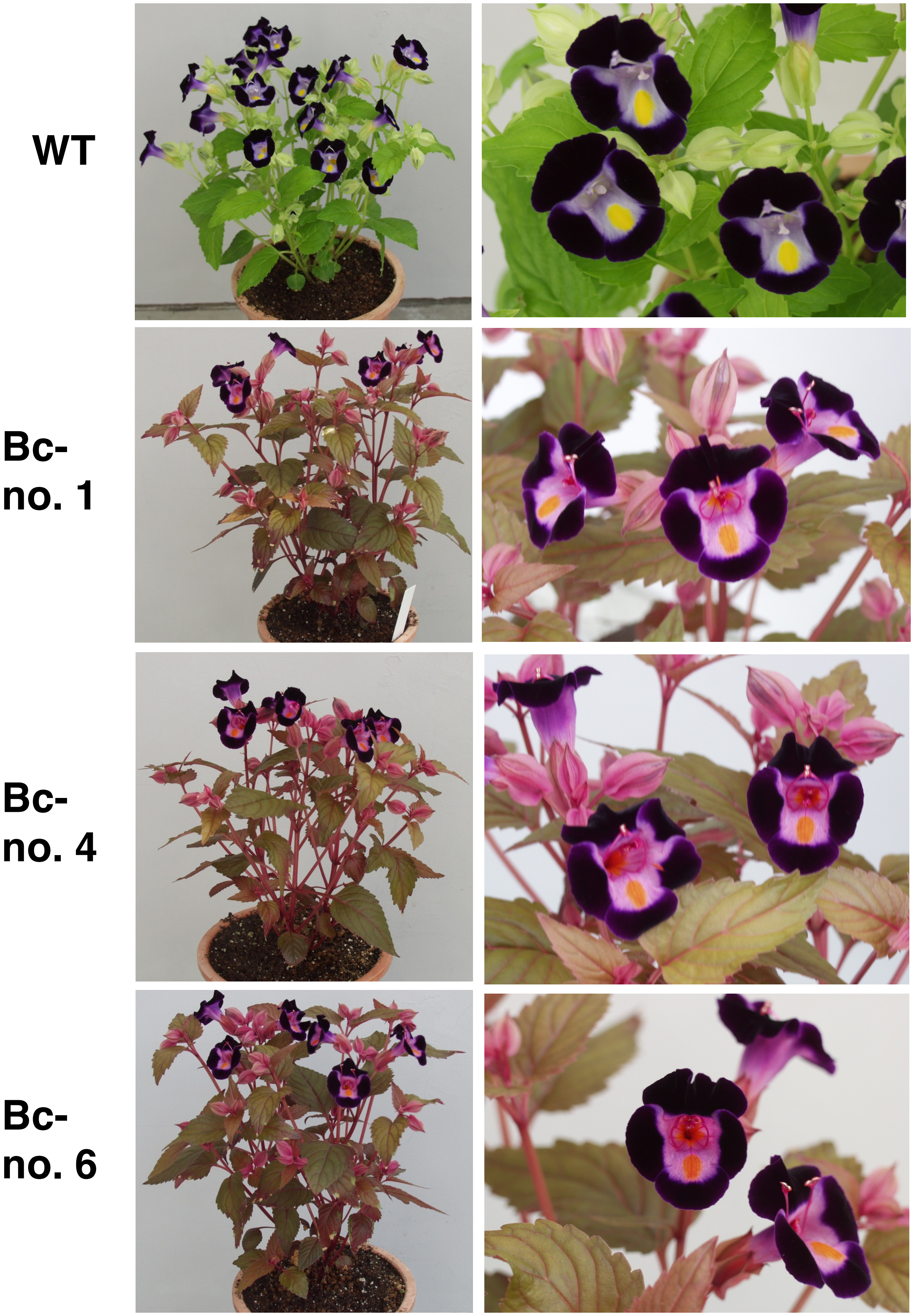

Supplementary Figure S4
Pictures of WT and transgenic torenia plants grown in a closed greenhouse.

Left panels present potted plants, and right panels present magnified flowers. The external diameter of the pots is about 13 cm.

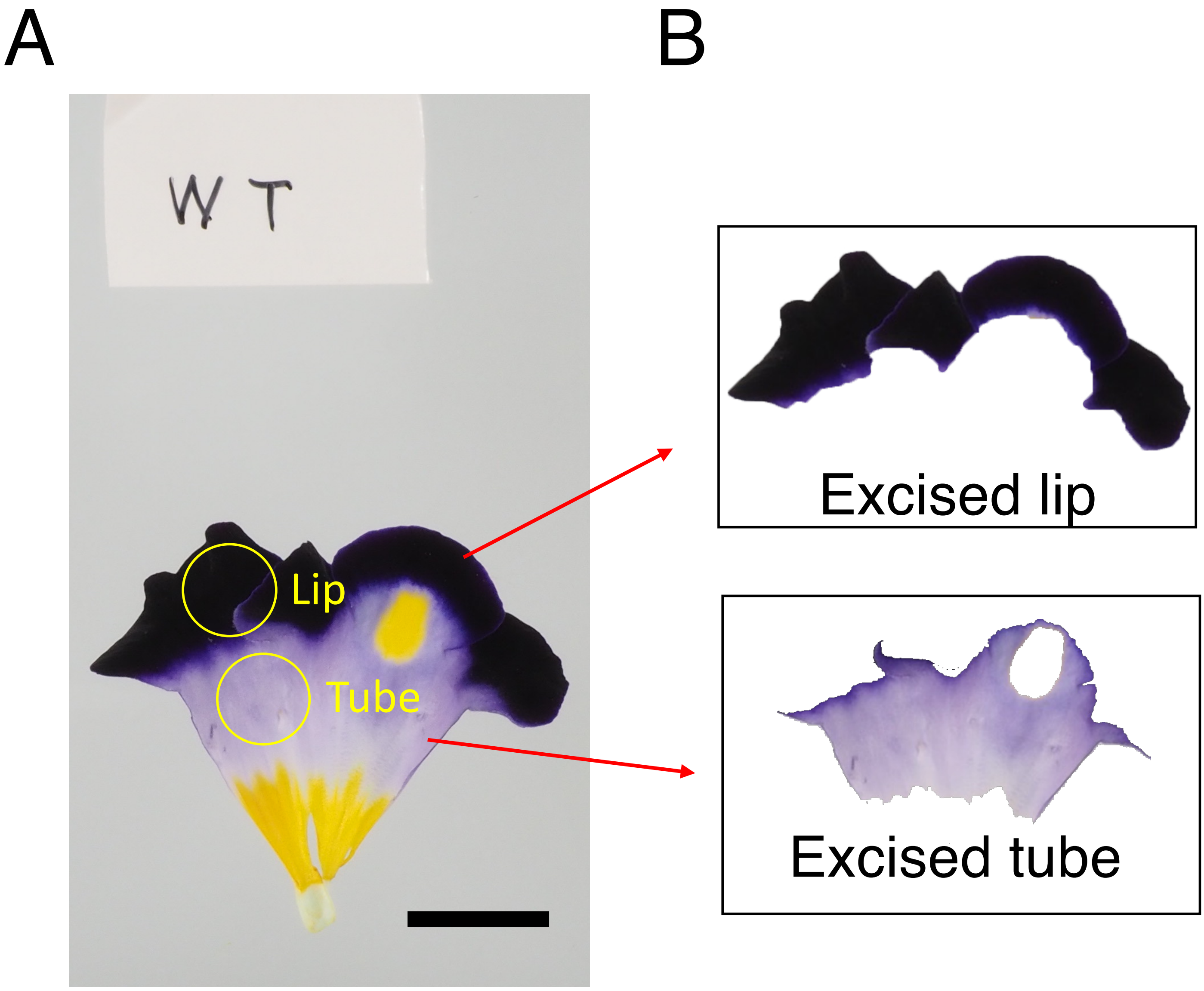

Supplementary Figure S5.
Petal parts used for spectrophotometric colorimetry and HPLC analysis.

(A) The image presents an example of flower from the WT plant. Yellow circles indicate lip and tube areas used for spectrophotometric analysis. Scale bar = 1 cm.

(B) Petal lip and tube parts were excised by hand as illustrated and subjected to HPLC analysis. Yellow regions containing carotenoids were removed from the analysis.

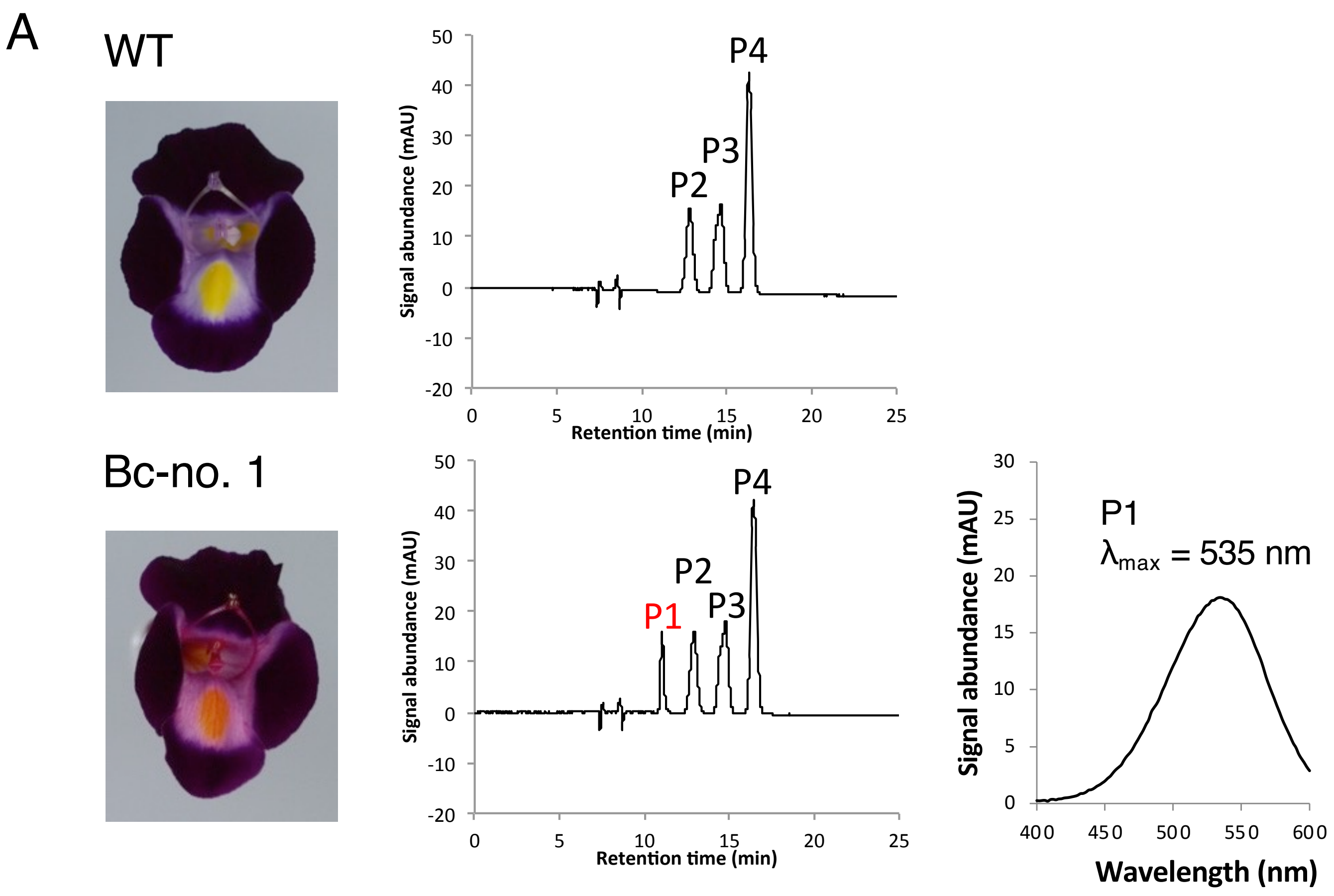

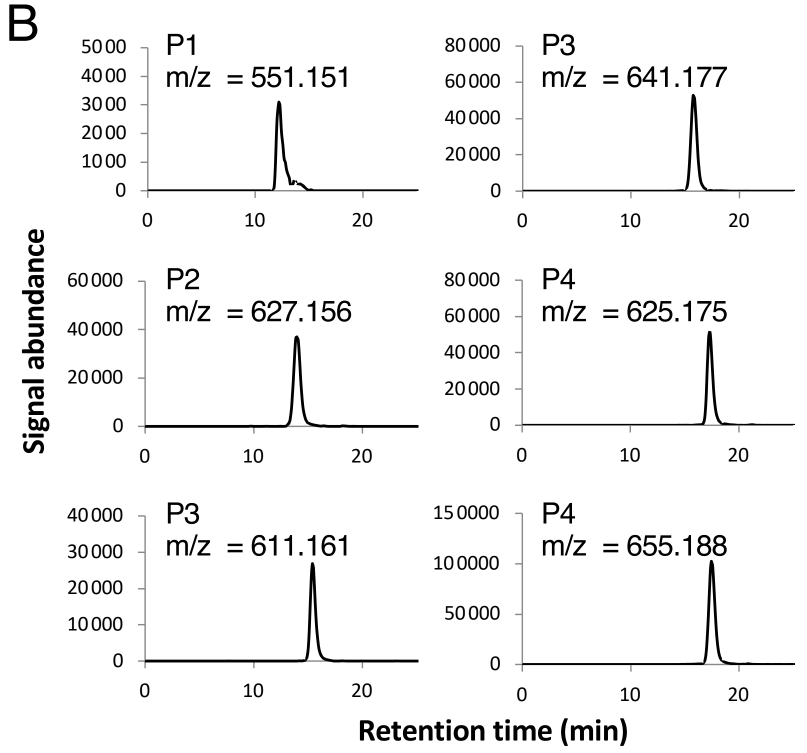

Peak	m/z	Compound
P1	551.151	Betanin<br>Isobetanin
P2	627.156	Del-3,5-G
P3	611.161	Cya-3,5-G
	641.177	Pet-3,5-G
P4	625.175<br>655.188	Peo-3,5-G<br>Mal-3,5-G

Supplementary Figure S6. LC-DAD-MS analysis of petals in WT and transgenic torenia plants.

Betacyanins and anthocyanins were analyzed with liquid by LC-DAD-MS.
(A) LC-DAD chromatograms at 535 nm. An additional peak P1 (λ max at 535 nm), corresponding to betacyanins, was detected in the transgenic line.
(B) LC-MS chromatograms of all detected compounds and predicted pigments.
